# Novel QUEST MRI *In Vivo* Measurement of Noise-induced Oxidative Stress in the Cochlea

**DOI:** 10.1038/s41598-019-52439-4

**Published:** 2019-11-07

**Authors:** André Kühl, Angela Dixon, Mirabela Hali, Aaron K. Apawu, Antonela Muca, Moaz Sinan, James Warila, Rod D. Braun, Bruce A. Berkowitz, Avril Genene Holt

**Affiliations:** 1 Department of Ophthalmology, Visual, and Anatomical Sciences, Wayne State University School of Medicine, Detroit, Michigan USA; 2 John D. Dingell Veteran Affairs Medical Center, Detroit, Michigan USA

**Keywords:** Cochlea, Nervous system, Diagnostic markers, Imaging techniques

## Abstract

Effective personalized therapeutic treatment for hearing loss is currently not available. Cochlear oxidative stress is commonly identified in the pathogenesis of hearing loss based upon findings from excised tissue, thus suggesting a promising druggable etiology. However, the timing and site(s) to target for anti-oxidant treatment *in vivo* are not clear. Here, we address this long-standing problem with QUEnch-assiSTed Magnetic Resonance Imaging (QUEST MRI), which non-invasively measures excessive production of free radicals without an exogenous contrast agent. QUEST MRI is hypothesized to be sensitive to noise-evoked cochlear oxidative stress *in vivo*. Rats exposed to a loud noise event that resulted in hair cell loss and reduced hearing capability had a supra-normal MRI R1 value in their cochleae that could be corrected with anti-oxidants, thus non-invasively indicating cochlear oxidative stress. A gold-standard oxidative damage biomarker [heme oxidase 1 (HO-1)] supported the QUEST MRI result. The results from this study highlight QUEST MRI as a potentially transformative measurement of cochlear oxidative stress *in vivo* that can be used as a biomarker for improving individual evaluation of anti-oxidant treatment efficacy in currently incurable oxidative stress-based forms of hearing loss.

## Introduction

Today, approximately 5% of the world’s population suffers from incurable noise-induced hearing loss acquired from a variety of noise conditions, including occupational exposures^1^, military duty–in and out of theater, and recreational activities. Such high noise exposures result in more hearing loss experienced earlier in life with life-long personal and societal costs^2^.

*Ex vivo* studies have implicated oxidative stress in the pathogenesis of noise-related hearing loss as well as in drug- and aging-related hearing loss^3–5^. Oxidative stress occurs when endogenous anti-oxidant levels are insufficient to reduce harmful interactions between the excessive production of free radicals and the surrounding tissue^4^. For example, exposure to loud noise generates reactive oxygen species (ROS) that permanently damage cochlear outer hair cells and the stria vascularis leading to loss of hearing^6,7^. Even a single noise exposure can have long term consequences with excessive free radical production persisting for up to ten days and causing lasting cochlear injury with decreased auditory sensitivity^8^. Importantly, in preclinical studies treatment with anti-oxidants given early in the course of the disease can prevent noise-induced hearing loss^9–11^. However, translating these results into effective treatments for people requires solving the long-standing problem that conventional assays cannot measure cochlear oxidative stress *in vivo*^12^.

A non-invasive biomarker for excessive free radical production has recently been developed suggesting a method to address this problem. The method, QUEnch-assiSTed (QUEST) MRI^13–15^, detects excessive free radical production *in vivo* without an exogenously administered contrast agent. Free radicals are paramagnetic agents and thus a potential MRI contrast agent. Too many free radicals produced in an asynchronous manner (e.g., during oxidative stress) will shorten MRI T1 (spin–lattice relaxation time), causing the spin–lattice relaxation rate, R1 (=1/T1), to increase in direct proportion to their concentration^16^. Reduction in R1 after anti-oxidant treatment (i.e., a quench) provides a non-invasive measure of oxidative stress^13–15^.

In this study, we test the hypothesis that harmful noise-induced oxidative stress can be measured *in vivo* using QUEST MRI^13–15^. Sprague Dawley rats were exposed to acoustic overstimulation to generate oxidative stress and were studied before and after anti-oxidant treatment with a combination of two anti-oxidants (AO’s): methylene blue (an alternative electron transporter that inhibits superoxide radical formation by mitochondria and oxidases) and α-lipoic acid (a free radical scavenger)^14,17,18^. The results were compared with a “gold standard” biomarker for oxidative stress: *ex vivo* whole mount preparations of the cochlea labeled for heme oxygenase I^19^; hair cell loss and hearing loss were also evaluated as other markers^20^.

## Results

### Acoustic trauma results in ABR threshold shifts and outer hair cell loss throughout the cochlea

**Figure 1:**
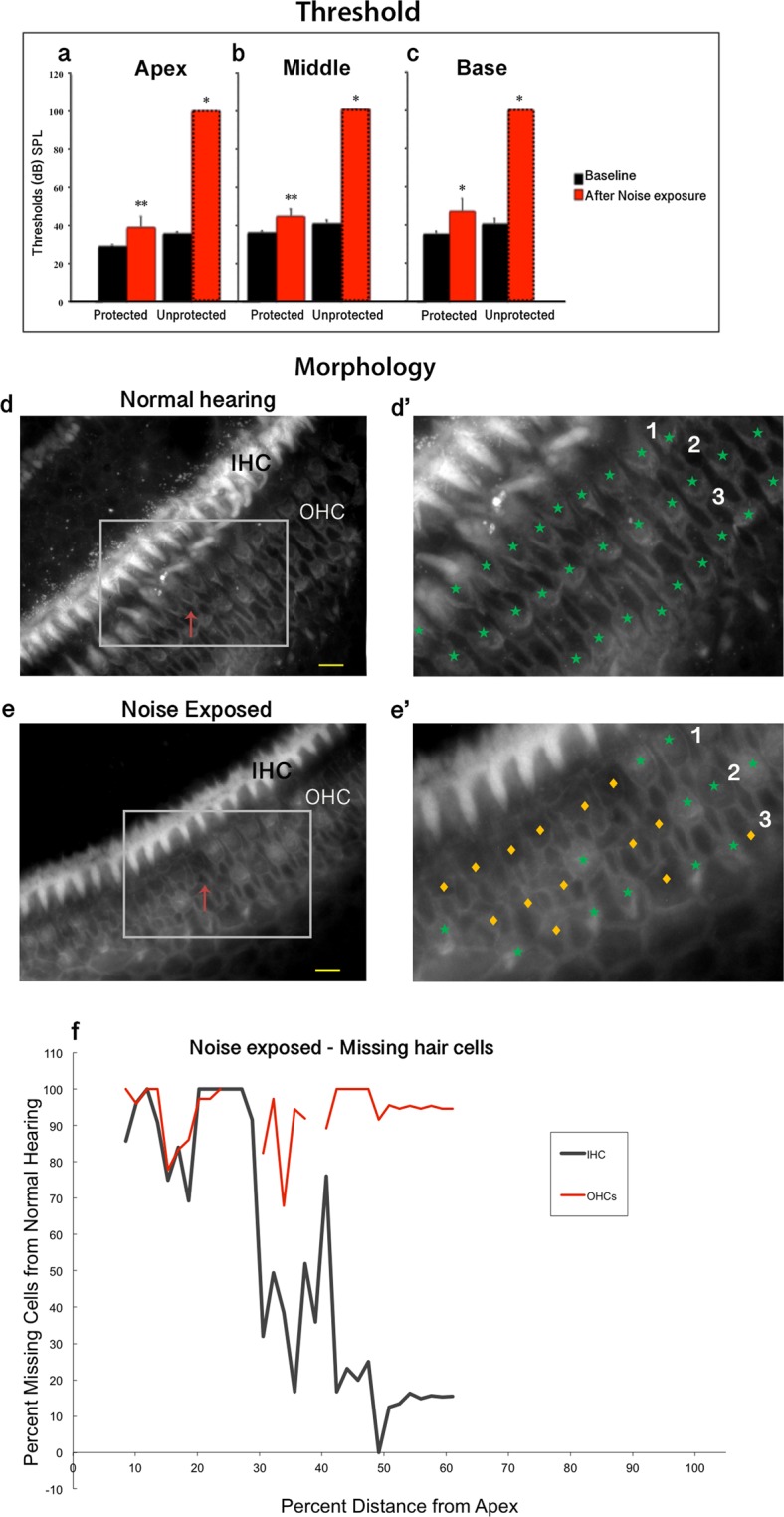
Comparison of hearing thresholds and cochlear epithelia from noise exposed and normal cochleae. (**a**–**c**) Auditory brainstem responses (ABRs) were compared at frequencies testing the apex (4 and 12 kHz), middle (20 and 25 kHz), and base of the cochlea (36 kHz), before (baseline – black bars) and after acoustic trauma (red bars) with protected and unprotected ears compared in each cochlear subdivision. Phalloidin staining of the cochlea (only apex shown) with present (green stars) and absent (gold diamonds) outer hair cells from normal hearing (**d**–**d’** red arrow) and noise exposed groups (**e**–**e’** red arrow). The resulting cytocochleogram reports the percent of missing hair cells (**f**). Error bars indicate standard error of the mean; Scale bar = 10 µm; 1–1^st^ row of outer hair cells, 2–2^nd^ row of outer hair cells, 3–3^rd^ row of outer hair cells. Asterisks - *p ≤ 0.05; **p ≤ 0.001.

To determine the impact of noise exposure on hearing sensitivity, hearing thresholds from silicone protected and unprotected cochleae were assessed using ABRs 48 hrs after the noise trauma. Baseline hearing thresholds in the protected ear were 29 ± 0.6 dB SPL (mean ± SEM) in the apex, 35 ± 0.8 dB SPL in the middle, and 35 ± 1.4 dB SPL in the base (Fig. 1a,b). Following acoustic trauma, the unprotected ear showed evidence of profound hearing loss with significant elevated thresholds (Fig. 1) of greater than 60 dB across the apex, middle, and base of the cochlea (p ≤ 0.05). Small, but significant elevations in hearing thresholds were also observed in the protected ear throughout the cochlea (apex - 10 ± 5 dB *p* = 0.001; middle - 9 ± 3 dB *p* = 0.001; base - 12 ± 7 dB *p* = 0.02).

To determine whether noise exposure resulted in inner (IHC) or outer (OHC) hair cell loss, phalloidin-stained hair cells were counted and compared across groups (Fig. 1d,e). The unprotected ear from NE rats revealed missing outer hair cells throughout all cochlear turns, from apex to base, while the cochleae from ears of animals in the *NH* group showed minimal hair cell loss (Fig. 1d–f). The loss of OHCs was greatest along the cochlear spiral 20–30% and 45–50% from the apex (89–100% absent). The IHCs showed a similar pattern, with the greatest loss occurring in the low frequency region of the cochlea (up to 30% from the cochlear apex). The results show that our noise exposure resulted in hair cell damage and hearing loss primarily in the apex and middle regions of unprotected cochleae.

### Only cochleae from the NE group demonstrate elevated R1, which is corrected with antioxidants

**Figure 2:**
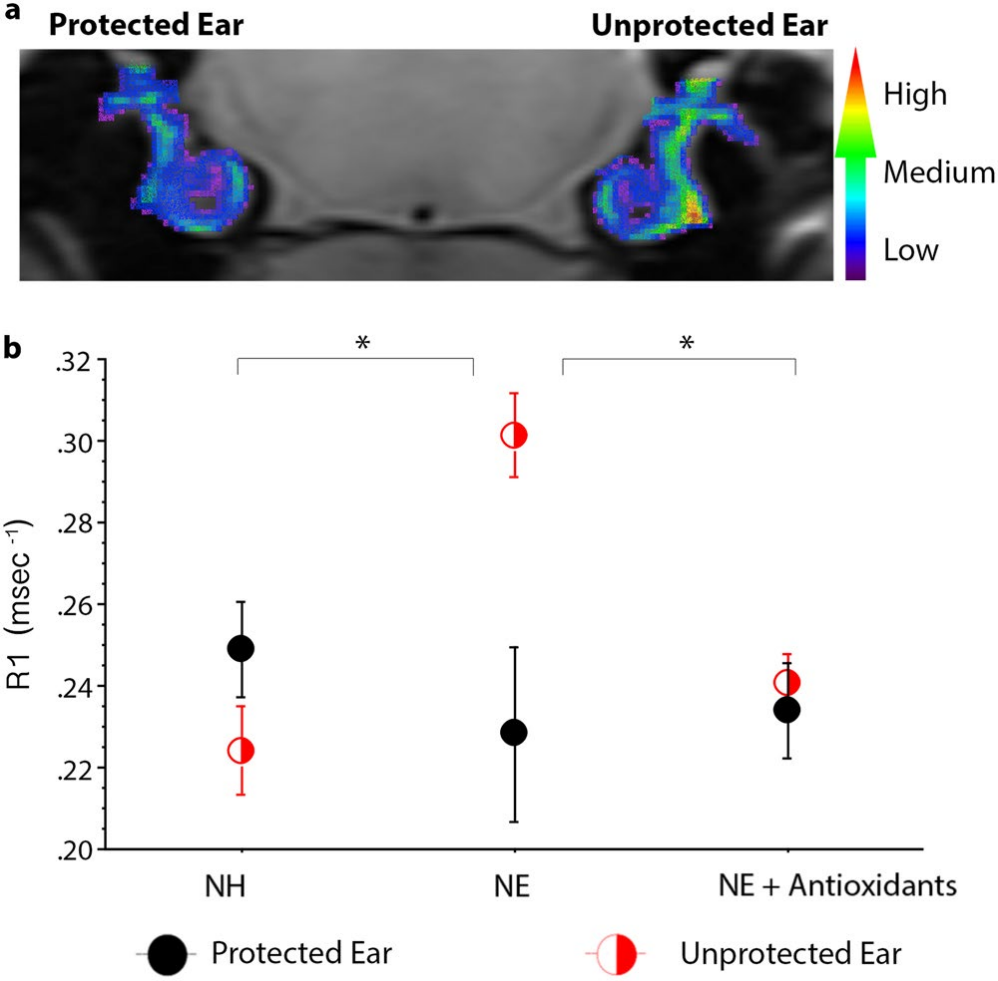
Noise-evoked cochlear oxidative stress measured *in vivo* with QUEST MRI. R1 maps were generated and used to compare protected and unprotected cochleae across groups (**a**). Mean change in R1 values from the protected ear in the NH group are displayed for each ear in all groups (**b**). Scale bar in (**a**) is a graded colorimetric representation of R1 values from high values represented in red to low values represented in blue. Error bars indicate standard error of the mean; Asterisks (*) = *p* ≤ 0.05.

Next, we examined noise-protected and unprotected cochleae for an increased R1 that could be corrected with antioxidants *in vivo* as an indicator of oxidative stress. QUEST MRI R1 maps were generated in a cross-sectional design (Fig. 2a). When R1 values were compared to animals in the NH group [0.236 ± 0.009] the unprotected ears of animals in the NE [0.301 ± 0.010] group had values that were significantly elevated (Fig. 2b; *p* = 0.01), suggestive of excessive free radical production. The unprotected ears of animals in the NE + AOs group [0.240 ± 0.007] displayed no significant difference (*p* = 0.71) when compared to the animals in the NH group. In contrast, when unprotected ears from the NE group were compared to those from the NE + AOs group, there was a significant decrease (*p* = 0.003) in R1 values. In all groups, ears protected by the silicone plug showed no significant differences in R1 values [NH - 0.249 ± 0.012; NE - 0.228 ± 0.021; NE + AOs - 0.234 ± 0.012; *p* = 0.11]. A two-way ANOVA with Scheffé post hoc testing was used with treatment (NH, NE, or NE + AO) and ear (protected or unprotected) as factors. Treatment and ear as factors were significant (*p* = 0.01 and *p* = 0.002). There were also significant between factor interactions (*p* = 0.004). The results are consistent with noise-evoked excessive free radical production within the cochlea *in vivo*.

### Noise-induced increase in heme oxygenase-1 (HO-1) levels in the organ of corti is blocked by anti-oxidants

**Figure 3:**
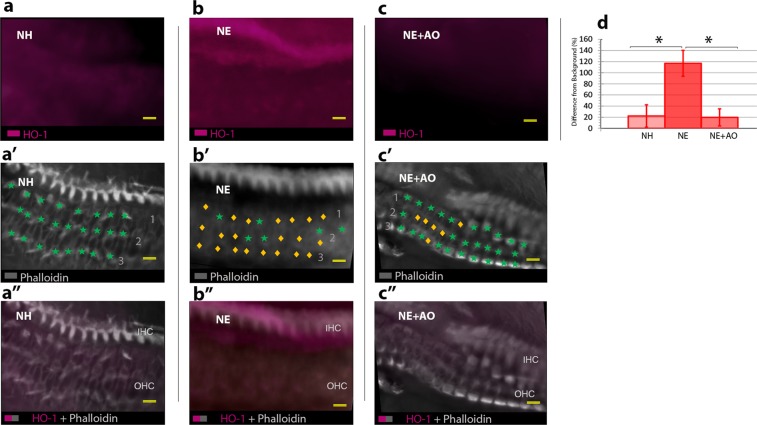
Noise exposure increases HO-1 immunolabeling levels in the cochlea, but the increase is reduced by anti-oxidant administration. Labeling for HO-1 (red) is observed in the apex of cochleae from NH controls (**a,a”**), NE (**b,b”**), and NE + AO (**c,c”**). Phalloidin staining was used to visualize anatomical structure in the NH (**a’**), NE (**b’**), and NE + AO (**c’**) groups. Scale bar = 10 μm. The percent change in HO-1 intensity from background (**d**) was assessed across groups (NH = normal hearing, NE = noise exposed, NE + AO = NE + anti-oxidants; IHC – inner hair cell, OHCs = outer hair cells; HO-1 = heme oxygenase-1; green stars denote presence of OHCs; gold diamonds denote absence of OHCs; 1–1^st^ row of outer hair cells, 2–2^nd^ row of outer hair cells, 3–3^rd^ row of outer hair cells. Error bars indicate confidence intervals; Asterisks = *p* ≤ 0.05).

As further validation of the QUEST MRI results for oxidative stress following noise exposure with and without anti-oxidant administration, we performed immunocytochemistry for HO-1 in the apex of the cochlea (Fig. 3), a “gold-standard” oxidative stress assay^21,22^. The apex was selected for analysis, since the most severe hair cell damage and hearing loss occurred primarily in the apex of unprotected cochleae (Fig. 1f). Changes in HO-1 immunolabeling intensity are reported as a percent change from the background level, calculated as (HO-1 – background)/background. A mixed-factor ANOVA was fit with random intercepts for each rat and individual error variances for left and right ears. The effect for treatment was significant (*p* = 0.0005, Fig. 3a–d). In addition, *post-hoc* comparisons of least-squares means^23^ showed that the intensity of HO-1 labeling in the Organ of Corti from noise-exposed animals (119.1 ± 12.2%) was significantly elevated compared to that from normal hearing animals (21.7 ± 19.4%), for a difference of 97.4% (*p* = 0.0006). The difference above background for the antioxidant group (20.4 ± 15.9%) was not significantly different from that for the control group (21.7 ± 19.4%; *p* = 0.98). These results confirm noise-stimulated excessive free radical production in the cochlea identified non-invasively with QUEST MRI.

## Discussion

In this study, using a model of noise-induced hearing loss, we found, for the first time *in vivo*, cochlear oxidative stress as measured by QUEST MRI in agreement with a gold-standard *ex vivo* assay. The QUEST MRI method is thought to be an integrative index representing the sum of continuous production of several species of free radicals^24^, and thus is a potentially useful global readout of free radical burden after auditory injury where the sources and species of free radicals likely vary over time. Future studies will focus on targeting and extinguishing particular sources of excessive free radical production (e.g., mitochondria, peroxisome, nicotinamide adenine dinucleotide phosphate oxidase) and/or species (e.g., superoxide free radical, hydroxyl free radical, nitric oxide) with a combination of targeted anti-oxidant therapies and genetically modified animal models. Other MRI methods can evaluate aspects of oxidative stress *in vivo* but usually require either highly specialized, nonstandard equipment (e.g., electron paramagnetic resonance imaging), or injection of stable free radical–sensitive reporter contrast agents. Instead, QUEST MRI simply requires two scans obtained before and after acute administration of FDA-approved anti-oxidants. The present results set the stage for potentially transformative advances over conventional assays that can only be performed on post-mortem tissue. Thus, QUEST MRI has high translational potential to improve pre-clinical and clinical evaluation of devastating oxidative stress-based hearing loss linked to noise, drug-use, or aging in order to guide management of prodromal anti-oxidant intervention and treatment.

### A model of noise-induced hearing loss with permanent hearing threshold shifts results from cochlear injury

We have used auditory brainstem responses and cytocochleograms to assess the degree of cochlear damage produced by the noise exposure paradigm used in the current study.

**Figure 4:**
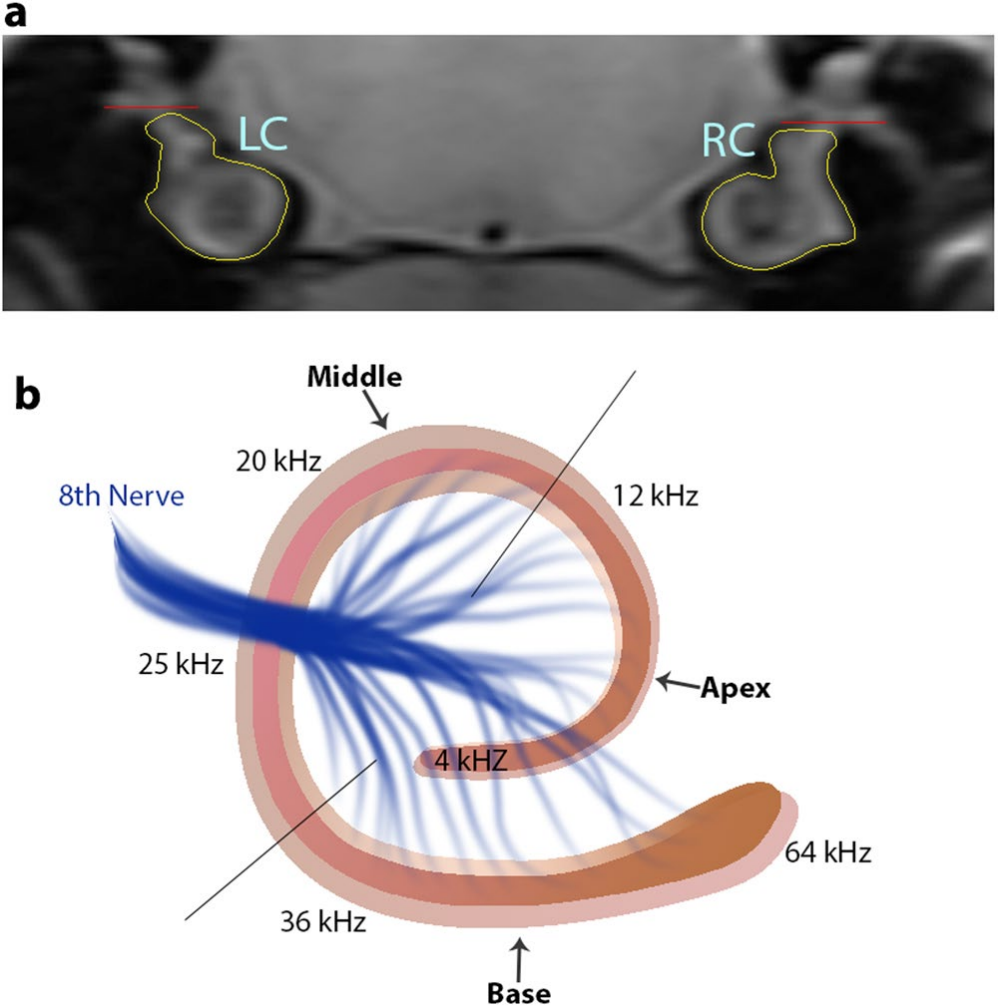
Cochlear regions of interest (**a**) - Identification of regions of interest for T1 analysis of the cochlea. A 400 µm scan through the cochlea (caudal- rostral view) was obtained using specific anatomical landmarks to maintain consistency between animals. The region of interest represents the entire cochlea in this plane of section (yellow outline) viewed from caudal to rostral. Each region was ventral to and did not include the vestibular system (dorsal to red line). LC = Left Cochlea; RC = Right Cochlea. (**b**) Schematic of the RC viewed from a caudal perspective comparable to the MRI image in panel a. The representation of frequency within dissected cochlear subdivisions; apex for low frequencies, middle for intermediate frequencies, and base for high frequencies. Lines delineate where cuts were made to divide the cochlea for immunocytochemistry and staining.

By using tone pips to test hearing responses at five specific frequencies, (4–36 kHz), specific regions of the cochlea were assessed and correlated with regions in the cochlea where hair cells respond best to sound of different frequencies; hair cells located in the base are more responsive to high frequency sounds, while hair cell in the apex are most responsive to low frequency sounds (Fig. 4). In accordance with our previous studies using this same noise exposure paradigm, hearing thresholds were significantly elevated across tested frequencies^25–27^. Thus, as expected, hair cell loss was most extensive for outer hair cells, with moderate to large percentages of absent cells found in each cochlear turn (Fig. 1). While in the current study only male rats were used, in the future female rats should be incorporated  since there are reports of differences in male and female susceptibility to noise induced hearing loss^28–30^ and likely oxidative stress^31,32^. For inner hair cells the majority of loss was found in the apex of the cochlea. These results are reasonable given that the employed noise exposure paradigm produced the greatest energy at low frequencies, which therefore produced the greatest injury in the apex of the cochlea. Overall, we demonstrate production of the expected injury in the expected cochlear location for this noise exposure paradigm.

### Prolonged loud noise results in pathogenic free radical levels and cochlear injury

Inner and outer hair cell death following loud noise exposure has been suggested to occur as a consequence of excessive free radical production based on *ex vivo* studies of the Organ of Corti and the induction of necrotic and apoptotic hair cell death pathways^20^. Noise-precipitated loss of hair cells as a result of oxidative stress has been reported to be a cause of threshold shifts observed after noise-induced hearing loss^4,33^. While the exact mechanism by which oxidative stress leads to neurodegeneration remains unclear, the mitochondria are likely involved^34^.

The mitochondrial electron transport chain is considered a major source of reactive oxygen species (ROS) produced by all tissues including those of the cochlea. Under normal physiological conditions, 1–2% of molecular oxygen is reduced to superoxide. In the cochlea following noise exposure however, the mitochondrial electron transport chain of hair cells is known to use large amounts of oxygen due to increased metabolic activity that results in the excess generation of reactive oxygen species^4^. This noise induced oxidative damage has been well documented and seen throughout cochlear fluids and tissues, and includes the formation of other free radicals in the form of reactive nitrogen species derived from nitric oxide as well^8,33^. Non-invasive measures of excessive free radical production within the cochlea would provide key information about cochlear injury and the effectiveness of therapeutic intervention. The biggest obstacle to-date in evaluating free radical levels in patients is that the majority of free radical assessment methods are based on *ex vivo* assays from the tissue of interest. Therefore, this study addresses a long-standing need for a reliable method that allows *in vivo* assessment of cochlear oxidative stress and can guide translation of effective anti-oxidants from the lab to the clinic.

### Cochlear free radical production can be assessed *ex vivo* with HO-1

In this study, we detect the presence of excessive free radical production in noise-exposed animals using both a novel *in vivo* technique, QUEST MRI, and the gold-standard assay for oxidative damage (*ex vivo* labeling for HO-1). Heme oxygenase is an enzyme responsible for the rate-limiting step in heme degradation, and elevation of HO-1 is widely accepted as a biomarker for oxidative stress since the protein is induced during excessive free radical production^35^.

The heme oxygenase-1 enzyme is one of two genetically distinct isozymes that is expressed under conditions of oxidative stress and indicates the activation of an endogenous anti-oxidant system^36^. In previous studies, two oxidative stress markers, HO-1 and 8-isoprostane (lipid peroxidation), were elevated in the cochlea following exposure to a 115 dB SPL octave band sound for 5 hrs^37–39^. We therefore predicted that in our model, exposure to similar noise levels would lead to increased production of HO-1 within the cochlea. Our results support this hypothesis, since 48 hours after acoustic trauma, immunocytochemistry in the ears of noise exposed animals revealed significantly elevated intensity levels for HO-1. While other groups have demonstrated the highest levels of noise induced HO-1 labeling in the OHCs^22,38^, our results indicate higher levels of HO-1 labeling in IHCs. Our findings could be the result of several factors, including species differences since previous studies used guinea pigs and the current study used rats. Also, the noise exposure in the current study resulted in a permanent threshold shift with a large appreciable loss of OHCs (Fig. 3b’). This could account for our result of less overall HO-1 labeling in the OHC region of the cochlea. Importantly, when compared to the other groups, the noise exposure + anti-oxidants group did not demonstrate elevated HO-1 intensity levels within the cochlea, further supporting the free radical quenching approach used in these studies. These results substantiate that not only does the noise exposure result in the expected injury in the expected location, but also that noise delivered under our conditions results in an oxidative stress response in the cochlea.

### Cochlear free radical production can be assessed *in vivo* with QUEST MRI

Currently, the detection of free radical production is primarily limited to *ex vivo* methods, such as the HO-1 assay. The ability to triage and determine when, and in what tissue, anti-oxidant treatment would be most beneficial in rescuing from the acute and chronic phases of a loud noise exposure necessitates an *in vivo* detection method for oxidative stress. MRI has been used extensively in research and medicine as a high-resolution tool capable of visualizing internal organs *in vivo*. The QUEST MRI index measures abnormally high production of paramagnetic free radicals as a greater-than-normal spin-lattice relaxation rate 1/T1 (R1) that can be corrected (returned to baseline values) in the presence of an anti-oxidant (i.e., a quench)^13–15^. In MRI, nuclear spins (usually protons) are brought to a higher energy state with a radiofrequency pulse. The spin–lattice relaxation time (“T1”) is a “life-time” measurement describing how quickly nuclear spins return back to their equilibrium position along the direction of the static magnetic field while releasing energy to their surroundings (the “lattice”). Thus the basis for excessive free radical production detection in previous studies, as well as the current study, is based upon the fact that asynchronous and continuous production of free radicals imparts a net, endogenous paramagnetic contrast mechanism that is suppressed with antioxidants^13,40,41^. Therefore, we investigated whether QUEST MRI would be a feasible method for detecting excessive free radical production *in vivo* within the cochlea following noise exposure. We observed an elevation in R1 values following noise exposure in unprotected ears of animals, using a design that produced elevated levels of the oxidative stress inducible protein HO-1. The quenching of R1 values following anti-oxidant administration critically links the elevation of R1 intensity values with the presence of noise-induced excessive free radicals in the cochlea. Agreement between QUEST MRI and other “gold-standard” assays has been demonstrated in other tissues, such as the retina and liver^14,15^. QUEST MRI for cochlear free radical detection is envisioned for future studies that explore the role of specific anti-oxidants as treatments for noise-induced hearing loss.

We used two anti-oxidants that operate via different mechanisms to decrease free radical levels in the cochlea following noise exposure. Specifically, administration of the anti-oxidant methylene blue has been shown to ameliorate superoxide radical formation by mitochondria^42^. The second anti-oxidant used in the current study, α-lipoic acid, acts as a potent scavenger of free radicals present in tissues^43,44^. Used individually these anti-oxidants have been shown to dampen excessive free radicals in the cochlea produced following a host of insults^10,45–47^ (e.g. noise, aging, and cisplatin). Therefore, in the current study our results demonstrate that this combination of anti-oxidants reduces the production of free radicals to pre-noise exposure levels as shown both by the reversal of a significant change in R1 values as well as by reduced HO-1 immuno-related intensity levels.

Since being implicated in the pathogeneses of noise-induced, chemical-induced, and age-related hearing loss, oxidative stress has long been a topic of great interest in the field of hearing research. While many studies have been able to use *ex vivo* methods to detect the formation of excessive free radicals, our study provides a first-time demonstration of a feasible MRI method for detection of excessive free radical production *in vivo* without the use of any exogenous contrast agents. This method may be useful to researchers studying oxidative stress in the cochlea and could be useful clinically in triaging potential treatments for noise-induced hearing loss. These results also support future application of QUEST MRI for other auditory related conditions with oxidative stress as a part of their pathogenesis. A theranostic approach^48^ may be combined with QUEST MRI so that anti-oxidants (i.e, methylene blue) can be targeted to specific regions of the cochlea during critical treatment windows with the effects monitored *in vivo*. Refinement of QUEST MRI for clinical application related to the cochlea would be particularly beneficial not only to those who are exposed to loud noise, but those with the potential to lose hearing after treatment for cancer with known ototoxic agents such as cisplatin, the most widely used drug with the greatest ototoxicity^42,49–53^.

As a powerful new paradigm, QUEST MRI is poised to bridge the gap between detection and treatment of cochlear damage resulting from oxidative stress. QUEST MRI is thus expected to accelerate the discovery of effective treatments and guide application and assessment of existing therapies for oxidative stress related sensorineural hearing deficits.

## Materials and Methods

### Ethics statement

All animals were maintained in a Wayne State University (WSU) AAALAC accredited animal facility under the Division of Laboratory Animal Resources. Animals were treated in accordance with the Animal Welfare Act and DHHS “Guide for the Care and Use of Laboratory Animals”. All procedures were approved by the Wayne State University Institutional Animal Care and Use Committee (IACUC) in accordance with National Institutes of Health (NIH) guidelines.

### Subjects

A total of 30 specific pathogen-free adult male Sprague Dawley rats were obtained from Charles River Laboratories (Wilmington, MA) and used in this study. Animals were housed in cages in a vivarium with controlled temperatures (24 ± 1 °C) and light cycles (12 hours light and 12 hours dark). The animals were fed a standard diet and provided water *ad libitum* throughout the experimental protocol. Three groups of animals were assessed: *Normal Hearing* (NH) n = 11, *Noise Exposed* (NE) n = 11, and *Noise Exposed* + *Anti-oxidants* (NE + AOs) n = 8.

### Noise exposure

Just prior to noise exposure the left ear of each animal was protected by filling the ear canal with a silicone elastomer (Kwik Seal, World Precision Instruments, Sarasota, FL). Awake freely moving animals from the NE and NE + AOs groups were exposed to a 1/3 octave band noise centered at 10 kHz with a sound pressure level (SPL) of 118 dB generated using software (Daqarta v 10.3) and delivered by overhead speakers for four hours. The silicone plug was removed immediately following the noise exposure. Animals in the NE + AOs group were administered the anti-oxidants methylene blue and α-lipoic acid following the noise exposure per the procedure below. Animals in the NH group had one ear plugged for four hours, but were not exposed to loud noise or administered anti-oxidants.

### Anti-oxidant administration

For methylene blue and α-lipoic acid solutions saline was used as the vehicle and the pH was adjusted to 7.4. Methylene blue (1 mg/kg ip, 0.5 mg/ml) was administered 24 hours following noise exposure (i.e., 24 hours prior to MRI). For sources of free radicals not suppressed by methylene blue, the free radical scavenger, α-lipoic acid, was also administered to animals (50 mg/kg ip, 5 mg/ml) 48 hours after the noise exposure (i.e., one hour before MRI). During the one hour period just after administration of α-lipoic acid or saline, animals (all of which were singly housed) remained in their cages with filter tops and were placed in a quiet room with minimal ambient noise, to minimize excessive sound stimulation. Control NH and NE rats were given saline at both time points instead of methylene blue and α-lipoic acid.

### Auditory brainstem response

Auditory brainstem responses (ABRs) were measured before and after (24 and 48 hours) noise exposure. During ABR measurements, animals (n = 6) were anesthetized (75 mg/kg ketamine, 8 mg/kg xylazine, im) and placed in a sound attenuating booth. Subdermal reference electrodes were inserted below the ipsilateral pinna, active electrodes were placed on top of the head, and ground electrodes were placed below the contralateral pinna. A multi-field magnetic speaker (MF1,Tucker-Davis Technologies, Alachua, FL) was placed into the external ear canal of the test ear and 5 millisecond tone bursts were played during each of 1024 trials for each of five frequencies corresponding to function in the apex (4 and 12 kHz), middle (20 and 25 kHz), and base (36 kHz) of the cochlea. Sound was generated and the resulting evoked potentials were recorded, amplified, and filtered using software and hardware from Tucker-Davis Technologies (BioSigRZ version 5.7.0/2014, RZ6 Multi I/O Processor, SA1 Stereo Power Amp, RP2.1 Enhanced Real-Time Processor, RA4PA Medusa PreAmps, RA4LI Headstages, Tucker-Davis Technologies, Alachua, FL).

### MRI testing

Animals (n = 15) were imaged 48 hours after noise-exposure. Isoflurane was administered (5% for induction and 2% for maintenance) with room air as the vehicle to immobilize the animals during imaging. For the duration of the scan, animals with no silicone ear plugs, were placed and remained on a heated re-circulating water pad to maintain body temperature. High-resolution MRI scans were acquired using a 7 T Bruker Clinscan system with a receive-only 4 element coil array centered on the animal’s head. Single spin-echo (echo time [TE] 11 ms; 20 × 20 mm^2^, matrix size 192 × 192; slice thickness 400 µm; in-plane resolution 104 μm) images (23) were acquired at different repetition times (TRs) in the following order (number of scans per TR in parentheses): 0.15 second (6), 3.50 seconds (1), 1.00 seconds (2), 1.90 seconds (1), 0.35 second (4), 2.70 seconds (1), 0.25 second (5), and 0.50 second (3). To compensate for reduced signal-noise ratios at shorter TRs, progressively more images were collected for averaging as the TR decreased. Rostral and caudal MRI T1 maps of the cochlea were obtained from animals in each experimental group (Fig. 4a). Rats were allowed to regain consciousness after MRI examination.

### Cochleae collection

Within 90 minutes following the MRI scan, animals were deeply anesthetized (2–5 ml ip; FatalPlus Vortech, Dearborn, MI) and cochleae were dissected after transcardial perfusion (10% sucrose rinse followed by 4% paraformaldehyde). Following transcardial fixation, the cochleae were removed, and slowly perfused locally via the round window (4% paraformaldehyde). The cochleae were incubated for 30 minutes with 8% EDTA and immersed in 30% paraformaldehyde for 60 minutes.

### Microdissection of the cochlea

Following post-fixation, microdissection of the cochleae was performed to expose auditory epithelia. The bony capsule of the cochlea was first removed followed by removal of lateral wall tissues, including the spiral ligament and stria vascularis. Then, the tectorial membrane was removed from base to apex, allowing the removal of the modiolus with the attached Organ of Corti from the temporal bone. The Organ of Corti was then separated into three parts corresponding to the apex, middle, and base of the cochlea (Fig. 4b). The epithelia were then rinsed in PBS (4° C) in preparation for immunocytochemistry.

### Immunocytochemistry

Following microdissection, immunocytochemistry was performed (n = 7). Epithelia were placed in a blocking solution of 3% normal donkey serum (NDS; Jackson ImmunoResearch, West Grove, PA) for one hour on a shaker at room temperature (3:100 NDS in Phosphate-buffered saline (PBS) with 0.5% Triton X-100, Sigma chemical, St. Louis, MO). Subsequently, cochlear epithelia were incubated with mouse anti-Heme Oxygenase-1 (Thermo Fisher Scientific, Waltham, MA) 1:100 in PBS with 0.3% Triton X-100 (Sigma chemical, St. Louis, MO) for 48 hours (4 °C) followed by rinsing with PBS (60 minutes) and incubation with Alexa Fluor 594 donkey anti-Mouse IgG H + L (1:400 in PBS, Molecular Probes, Eugene, OR) for 1 hour.

### Cytochemical staining

Cytochemical staining was performed after immunocytochemistry to allow visualization of the hair cells. Cochlear epithelia were rinsed (PBS 2X for 30 minutes each) and incubated with a fluorophore conjugated phalloidin stain (Molecular Probes Invitrogen, Carlsbad, CA) at room temperature (1:100 in PBS 90 minutes) on a shaker in the dark. The tissues were then rinsed (2 × 30 minutes each).

### Analysis

#### Auditory brainstem responses

Evoked potentials were collected at frequencies of 4, 12, 20, 25 and 36 kHz with sound intensity levels beginning at 100 dB SPL and decreased in increments of 5 or 10 dB until a response was no longer elicited. Thresholds at each frequency were determined as the minimum sound level at which a wave 1 ABR response was still elicited.

#### MRI

Two 400 µm MRI images were collected through the cochlea. For the first scan, an image of the cochleae was collected in the same plane as that of the inferior colliculus and was designated as the rostral cochlear section. The next section through the cochleae was taken in a plane containing the cochlear nucleus using the shape of the fourth ventricle as a guide. The section was designated as the caudal cochlear section. After applying customized macros, ImageJ (Version 1.48p, Wayne Rasband National Institutes of Health, USA) was used to manually draw the regions of interest onto each image, acquired from rostral to caudal (viewed from caudal perspective) from the entire cochlea on T1 map images. Then, R1 was measured from the right and left cochleae for each subject (Fig. 4a). Briefly, the values were collected as follows:

Within each T1 data set 23 images were collected. Images acquired with the same TR were first registered (rigid body) and then averaged to generate a stack of 8 images. These averaged images were then registered across TRs. Using imperfect slice profiles leads to a systematic underestimate of T1 values [Chapter 18 in]^54^. This bias is corrected by normalizing to the shorter TR which results in a more accurate T1 estimate (Dr. E.M. Haacke, private communication). Specifically, first a 3 × 3 Gaussian smoothing filter (performed three times) was applied only on the image set acquired at the 150 ms TR in order to minimize noise, emphasize signal, and act as a low pass filter to infer the B1 map and account for RF coil inhomogeneities. The smoothed TR 150 ms image was then divided into the rest of the images of that T1 data set. Preliminary experiments (not shown) found that this procedure substantially helps minimize the day-to-day variation in the R1 profile previously noted and obviated the need for a “same-day” control group used previously for correcting day-to-day variations^13,14^. R1 maps were calculated using the seven normalized images via fitting to a three-parameter T1 equation (intensity = a + b*(exp(-c*TR)), where a, b, and c are fitted parameters) on a pixel-by-pixel basis using in-house R scripts. In this equation c = R1 = 1/T1.

### Immunocytochemistry and phalloidin staining

The specificity of the HO-1 antibody and the use of HO-1 as an oxidative stress biomarker have been validated and characterized previously^19,55^. Quantitative immunocytochemistry has previously been used to study auditory pathways^56,57^. Similar methods were used in the current study. Images of dissected tissue from the apex, middle, and base of the cochlea were acquired using a *Leica* microscope (DM 5000 wide-field microscope). For each subdivision of the cochlea (apex, middle, and base), three images were obtained. Each image covered a region encompassing 10 to 13 serial hair cells from low to high frequency along the cochlear spiral. In addition to counting the number of missing hair cells, the intensity of HO-1 immunolabeling in the epithelium was normalized to background regions containing no epithelium and compared across cochlear subdivisions and experimental groups (ImageJ, Version 1.48p, Wayne Rasband National Institutes of Health, USA). The exposure time was kept constant across slides within and across groups during acquisition of all HO-1 related fluorescent images.

### Statistical analysis

Statistical analyses were conducted using StatView (Version 5.0) and R 3.2.3 using the nlme package^58,59^. A two-way analysis of variance (ANOVA) was used to explore main effects and interactions within and between groups. Significance was defined as *p* ≤ 0.05. Post hoc comparisons were used to determine the direction of observed changes.

## Data Availability

Data generated in the present study are available from the corresponding author upon reasonable request.

## References

[1] World Health Organization. *Deafness and hearing loss*, http://www.who.int/news-room/fact-sheets/detail/deafness-and-hearing-loss (2018).

[2] Henderson, E., Testa, M. A., & Hartnick, C. Prevalence of Noise-Induced Hearing-Threshold Shifts and Hearing Loss Among US Youths. *Pediatrics* **127**, 10.1542/peds.2010-0926d (2010). 10.1542/peds.2010-0926 21187306

[3] Yamasoba T (2013). Current concepts in age-related hearing loss: epidemiology and mechanistic pathways. Hear Res.

[4] Henderson D, Bielefeld EC, Harris KC, Hu BH (2006). The role of oxidative stress in noise-induced hearing loss. Ear Hear.

[5] Evans P, Halliwell B (1999). Free radicals and hearing. Cause, consequence, and criteria. Ann N Y Acad Sci.

[6] Kamogashira T, Fujimoto C, Yamasoba T (2015). Reactive oxygen species, apoptosis, and mitochondrial dysfunction in hearing loss. Biomed Res Int.

[7] Ohlemiller KK, Wright JS, Dugan LL (1999). Early elevation of cochlear reactive oxygen species following noise exposure. Audiol Neurootol.

[8] Yamashita D, Jiang HY, Schacht J, Miller JM (2004). Delayed production of free radicals following noise exposure. Brain Res.

[9] Souza M, Costa K, Vitorino PA, Bueno NB, Menezes PL (2018). Effect of antioxidant supplementation on the auditory threshold in sensorineural hearing loss: a meta-analysis. Braz J Otorhinolaryngol.

[10] Park JS, Jou I, Park SM (2014). Attenuation of noise-induced hearing loss using methylene blue. Cell Death Dis.

[11] Ewert DL (2012). Antioxidant treatment reduces blast-induced cochlear damage and hearing loss. Hear Res.

[12] Sha SH, Schacht J (2017). Emerging therapeutic interventions against noise-induced hearing loss. Expert Opin Investig Drugs.

[13] Berkowitz BA (2016). MRI of Retinal Free Radical Production With Laminar Resolution *In Vivo*. Invest Ophthalmol Vis Sci.

[14] Berkowitz BA (2015). Measuring *In Vivo* Free Radical Production by the Outer Retina. Invest Ophthalmol Vis Sci.

[15] Stinnett, G. *et al*. A Novel Assay for the *In Vivo* Detection of Reactive Oxygen Species Using MRI. *ISMRM Meeting Abstracts*, 1917 (2015).

[16] Matsumoto K-i (2006). High-Resolution Mapping of Tumor Redox Status by Magnetic Resonance Imaging Using Nitroxides as Redox-Sensitive Contrast Agents. Clinical Cancer Research.

[17] Berkowitz BA (2017). *In vivo* imaging of prodromal hippocampus CA1 subfield oxidative stress in models of Alzheimer disease and Angelman syndrome. FASEB J.

[18] Berkowitz BA, Miller RA, Roberts R (2017). Genetically heterogeneous mice show age-related vision deficits not related to increased rod cell L-type calcium channel function *in vivo*. Neurobiol Aging.

[19] Waza AA, Hamid Z, Ali S, Bhat SA, Bhat MA (2018). A review on heme oxygenase-1 induction: is it a necessary evil. Inflamm Res.

[20] Furness DN (2015). Molecular basis of hair cell loss. Cell Tissue Res.

[21] Fetoni AR (2014). Curcuma longa (curcumin) decreases *in vivo* cisplatin-induced ototoxicity through heme oxygenase-1 induction. Otol Neurotol.

[22] Fetoni AR (2010). *In vivo* protective effect of ferulic acid against noise-induced hearing loss in the guinea-pig. Neuroscience.

[23] Lenth RV (2016). Least-Squares Means: The R Package lsmeans. Journal of Statistical Software.

[24] Berkowitz BA (2018). Oxidative stress measured *in vivo* without an exogenous contrast agent using QUEST MRI. J Magn Reson.

[25] Muca A (2018). Tinnitus and temporary hearing loss result in differential noise-induced spatial reorganization of brain activity. Brain Struct Funct.

[26] Fyk-Kolodziej BE (2015). Dopamine in the auditory brainstem and midbrain: co-localization with amino acid neurotransmitters and gene expression following cochlear trauma. Front Neuroanat.

[27] Holt AG, Bissig D, Mirza N, Rajah G, Berkowitz B (2010). Evidence of key tinnitus-related brain regions documented by a unique combination of manganese-enhanced MRI and acoustic startle reflex testing. PLoS One.

[28] McFadden, S. Sex Difference in Susceptibility and Resistance to Noise-induced Hearing Loss in Chinchillas. Report No. 20010323 024, 119 (The State University of New York at Buffalo Amherst, New York, 1998).

[29] Milon B (2018). The impact of biological sex on the response to noise and otoprotective therapies against acoustic injury in mice. Biol Sex Differ.

[30] Lauer AM, Schrode KM (2017). Sex bias in basic and preclinical noise-induced hearing loss research. Noise Health.

[31] Giordano G (2013). Gender differences in brain susceptibility to oxidative stress are mediated by levels of paraoxonase-2 expression. Free Radic Biol Med.

[32] Reimann K, Krishnamoorthy G, Wier WG, Wangemann P (2011). Gender differences in myogenic regulation along the vascular tree of the gerbil cochlea. PLoS One.

[33] Yuan H (2015). Autophagy attenuates noise-induced hearing loss by reducing oxidative stress. Antioxid Redox Signal.

[34] Kim GH, Kim JE, Rhie SJ, Yoon S (2015). The Role of Oxidative Stress in Neurodegenerative Diseases. Exp Neurobiol.

[35] Choi BM (2007). Piperine protects cisplatin-induced apoptosis via heme oxygenase-1 induction in auditory cells. J Nutr Biochem.

[36] Fetoni AR (2015). Rosmarinic acid up-regulates the noise-activated Nrf2/HO-1 pathway and protects against noise-induced injury in rat cochlea. Free Radic Biol Med.

[37] Roy S, Ryals MM, Van den Bruele AB, Fitzgerald TS, Cunningham LL (2013). Sound preconditioning therapy inhibits ototoxic hearing loss in mice. J Clin Invest.

[38] Matsunobu, T., Satoh, Y., Ogawa, K. & Shiotani, A. Heme oxygenase-1 expression in the guinea pig cochlea induced by intense noise stimulation. *Acta Otolaryngol Suppl*, 18–23 (2009). 10.1080/00016480902933056 19848234

[39] Ohinata Y, Miller JM, Schacht J (2003). Protection from noise-induced lipid peroxidation and hair cell loss in the cochlea. Brain Res.

[40] Bakalova R (2015). Magnetic Resonance Imaging of Mitochondrial Dysfunction and Metabolic Activity, Accompanied by Overproduction of Superoxide. ACS Chem Neurosci.

[41] Hyodo F (2008). Monitoring redox-sensitive paramagnetic contrast agent by EPRI, OMRI and MRI. J Magn Reson.

[42] Ruan S (1999). Attenuation of WAF1/Cip1 expression by an antisense adenovirus expression vector sensitizes glioblastoma cells to apoptosis induced by chemotherapeutic agents 1,3-bis(2-chloroethyl)-1-nitrosourea and cisplatin. Clin Cancer Res.

[43] Biewenga GP, Haenen GR, Bast A (1997). The pharmacology of the antioxidant lipoic acid. Gen Pharmacol.

[44] Haramaki N, Han D, Handelman GJ, Tritschler HJ, Packer L (1997). Cytosolic and mitochondrial systems for NADH- and NADPH-dependent reduction of alpha-lipoic acid. Free Radic Biol Med.

[45] Wilson, T., Omelcheko, I., Foster, S. & Nuttall, A. L. *Loud sound induced hearing loss is prevented by methylene blue*, 2013).

[46] Seidman MD, Khan MJ, Bai U, Shirwany N, Quirk WS (2000). Biologic activity of mitochondrial metabolites on aging and age-related hearing loss. Am J Otol.

[47] Rybak LP, Husain K, Whitworth C, Somani SM (1999). Dose dependent protection by lipoic acid against cisplatin-induced ototoxicity in rats: antioxidant defense system. Toxicol Sci.

[48] Apawu AK (2018). MRI compatible MS2 nanoparticles designed to cross the blood-brain-barrier: providing a path towards tinnitus treatment. Nanomedicine.

[49] Hazlitt RA, Min J, Zuo J (2018). Progress in the Development of Preventative Drugs for Cisplatin-Induced Hearing Loss. J Med Chem.

[50] Francis SP, Cunningham LL (2017). Non-autonomous Cellular Responses to Ototoxic Drug-Induced Stress and Death. Front Cell Neurosci.

[51] Shoji F (2000). Differential protective effects of neurotrophins in the attenuation of noise-induced hair cell loss. Hear Res.

[52] Gabaizadeh R (1997). Protection of both auditory hair cells and auditory neurons from cisplatin induced damage. Acta Otolaryngol.

[53] Gabaizadeh R, Staecker H, Liu W, Van De Water TR (1997). BDNF protection of auditory neurons from cisplatin involves changes in intracellular levels of both reactive oxygen species and glutathione. Brain Res Mol Brain Res.

[54] Haacke, E. M., Brown, R. W., Thompson, M. R. & Venkatesan, R. *Magnetic Resonance Imaging: Physical Principles and Sequence Design*. (Wiley, 1999).

[55] Kim J (2012). *In vivo* regulation of the heme oxygenase-1 gene in humanized transgenic mice. Kidney Int.

[56] Ling LL, Hughes LF, Caspary DM (2005). Age-related loss of the GABA synthetic enzyme glutamic acid decarboxylase in rat primary auditory cortex. Neuroscience.

[57] Abbott SD, Hughes LF, Bauer CA, Salvi R, Caspary DM (1999). Detection of glutamate decarboxylase isoforms in rat inferior colliculus following acoustic exposure. Neuroscience.

[58] R Core Team, https://www.R-project.org/ (2016).

[59] Pinheiro, J., Bates, D., DebRoy, S., Sarkar, D. & Team, a. R. C., http://cran.r-project.org/package=nlme (2016).

