# Publisher Correction: Novel QUEST MRI *In Vivo* Measurement of Noise-induced Oxidative Stress in the Cochlea

**DOI:** 10.1038/s41598-019-55312-6

**Published:** 2019-12-27

**Authors:** André Kühl, Angela Dixon, Mirabela Hali, Aaron K. Apawu, Antonela Muca, Moaz Sinan, James Warila, Rod D. Braun, Bruce A. Berkowitz, Avril Genene Holt

**Affiliations:** 1 Department of Ophthalmology, Visual, and Anatomical Sciences, Wayne State University School of Medicine, Detroit, Michigan USA; 2 John D. Dingell Veteran Affairs Medical Center, Detroit, Michigan USA

Correction to: *Scientific Reports* 10.1038/s41598-019-52439-4, published online 07 November 2019

In the original version of this Article, the author Avril Genene Holt was incorrectly indexed. This error has now been corrected.

